# Comparison of Demographics and Oxford Knee Score in Total Knee Arthroplasty Patients Between the United Kingdom and Saudi Arabia: A Cohort Study

**DOI:** 10.3390/jcm14124148

**Published:** 2025-06-11

**Authors:** Omar W. Althomali, Bodor Bin sheeha, David Sands Johnson, Zizi M. Ibrahim, Shaimaa Abdelhamid Altoury, Richard Jones

**Affiliations:** 1 Department of Physiotherapy, College of Applied Medical Sciences, University of Ha’il, P.O. Box 2240, Ha’il 81442, Saudi Arabia; 2 Department of Rehabilitation Sciences, College of Health and Rehabilitation Sciences, Princess Nourah bint Abdulrahman University, P.O. Box 84428, Riyadh 11671, Saudi Arabia; bhbinsheeha@pnu.edu.sa (B.B.s.); zmibrahim@pnu.edu.sa (Z.M.I.); 3 Department of Orthopaedics, Stockport NHS Foundation Trust, Stockport SK2 7JE, UK; david.johnson@stockport.nhs.uk; 4 Faculty of Physical Therapy, Cairo University, P.O. Box 12613, Giza 12611, Egypt; dr.shaimaa.altoury@gmail.com; 5 School of Health and Society, University of Salford, Salford M6 6PU, UK; r.k.jones@salford.ac.uk

**Keywords:** osteoarthritis, Oxford knee score, total knee arthroplasty, United Kingdom, Kingdom of Saudi Arabia, cultural variation, pain and function

## Abstract

**Background/Objectives**: Total knee arthroplasty (TKA) is considered as important final line of treatment for several conditions such as knee osteoarthritis. Interestingly, culture, demographics, and health care systems may differ between countries, leading to different outcomes. Understanding this variation can help in enhancing patient care and rehabilitation therapy. This study aimed to compare primary TKA patients from the Kingdom of Saudi Arabia (KSA) and the United Kingdom (UK) pre- and post-surgery, representing two different populations and cultural backgrounds. **Methods**: A retrospective cohort sample was collected from the UK and one prospective cohort sample was collected in the KSA. Demographic characteristics and the Oxford knee score (OKS) were compared preoperatively and 6 and 12 months postoperatively. **Results**: A total of 259 patients were included in the study. Significant differences were observed in demographic characteristics. Patients in the UK cohort were five years older and 7 kg/m^2^ lower in body mass index (BMI) than those in the KSA cohort. The proportion of male patients was higher in the UK cohort (37%) compared to the KSA cohort (17%). Preoperatively, the OKS was significantly (*p* = 0.001) worse in the Saudi cohort (15 ± 4) compared to the UK cohort (19 ± 6). After 6 months postoperatively, both groups improved; however, there was a significant difference, with a higher value for the KSA cohort compared to the UK cohort. By 12 months post-TKA, the difference in OKS between the populations no longer existed. **Conclusions**: Although there are notable differences in demographics and preoperative OKS, the functional outcomes at 12 months post-TKA were comparable between the two populations. These findings suggest that while cultural and demographic factors may influence early postoperative recovery, 12-month outcomes tend to converge across different populations.

## 1. Introduction

Total knee arthroplasty (TKA) is considered a widely performed procedure and one of the most successful surgical procedures for managing advanced knee osteoarthritis and other conditions, such as rheumatoid arthritis [1,2]. The procedure involves replacing the affected articular surfaces with artificial components [3]. TKA has been established as a successful treatment for patients whose symptoms are not adequately controlled by a conservative approach such as weight reduction, physical therapy intervention, or pharmacological modalities [3]. The operation plays an important role in enhancing the patient’s quality of life by enhancing mobility, improving joint function, and reducing chronic pain [2,4]. The demand for total knee replacement has increased with increasing prevalence of knee osteoarthritis in aging populations [3,5].

Supporting evidence has shown that the number of TKA procedures has significantly increased over the last few years. In the United States, the number of primary TKA annular procedures is anticipated to reach 3.5 million by 2030 [5]. In the United Kingdom, it has been projected that 35 thousand patients will undertake a total knee replacement every year [6,7,8]. In the Kingdom of Saudi Arabia, previous research has shown that the annual number of knee replacements has substantially increased in recent years [1].

The Oxford knee score (OKS) is a valid, reliable, and widely used patient-reported outcome designed to assess knee function and pain among individuals with TKA. OKS is particularly beneficial for assessing both preoperative and postoperative knee function, granting insights into surgical efficacy and patient satisfaction [9,10]. Studies have shown that OKS is highly sensitive to changes in knee function following TKA and has a satisfactory correlation with other assessment questionnaires such as the American Knee Society score (AKSS) and the Western Ontario and McMaster Universities osteoarthritis index (WOMAC) [11]. Due to its ease of use and reliability, OKS is commonly used in clinical studies to compare TKA results across different healthcare settings and populations [12].

Although TKA is a highly successful treatment, several factors may influence the outcomes, including geographical location, implant selection, comorbidities, and rehabilitation protocols [13,14]. Researchers have found that demographic characteristics substantially influence the success of TKA outcomes [15]. Furthermore, studies have found that factors such as age, gender, body mass index (BMI), and comorbidities can play a role in the surgical outcome [14,15]. Interestingly, obesity has been identified as being linked to several problems such as higher complication rates, infection, implant loosening, and reduction in the survival of the prosthesis [5]. Additionally, cultural and healthcare system differences can influence postoperative healing, rehabilitation, and patient satisfaction [16].

Cultural and socioeconomic background differences between the United Kingdom and the Kingdom of Saudi Arabia populations, along with differences in healthcare systems, may impact demographics and therefore affect TKA outcomes. In the UK, most patients undergoing TKA are female and over the age of 60 years old [17]. In the KSA, the ages range from 28 to 85 years old. Also, the percentage of females is more predominant, with studies showing that more than 77% of included TKA patients were female [1,18,19]. Furthermore, the frequency of diabetes among patients with TKA in the KSA is high (67%) which may impact outcomes [19].

Given these variations between the UK and the KSA, this study aims to compare TKA outcomes between patients in the two countries, considering differences in demography, culture, and healthcare systems. Understanding these factors will help to optimizing procedure and rehabilitation strategies for enhancing patient care and long-term functional outcomes. The goal was to explore the effect of demographic and cultural differences and healthcare systems throughout, comparing outcomes before and after surgery.

## 2. Materials and Methods

### 2.1. Study Design in the UK

**Figure 1:**
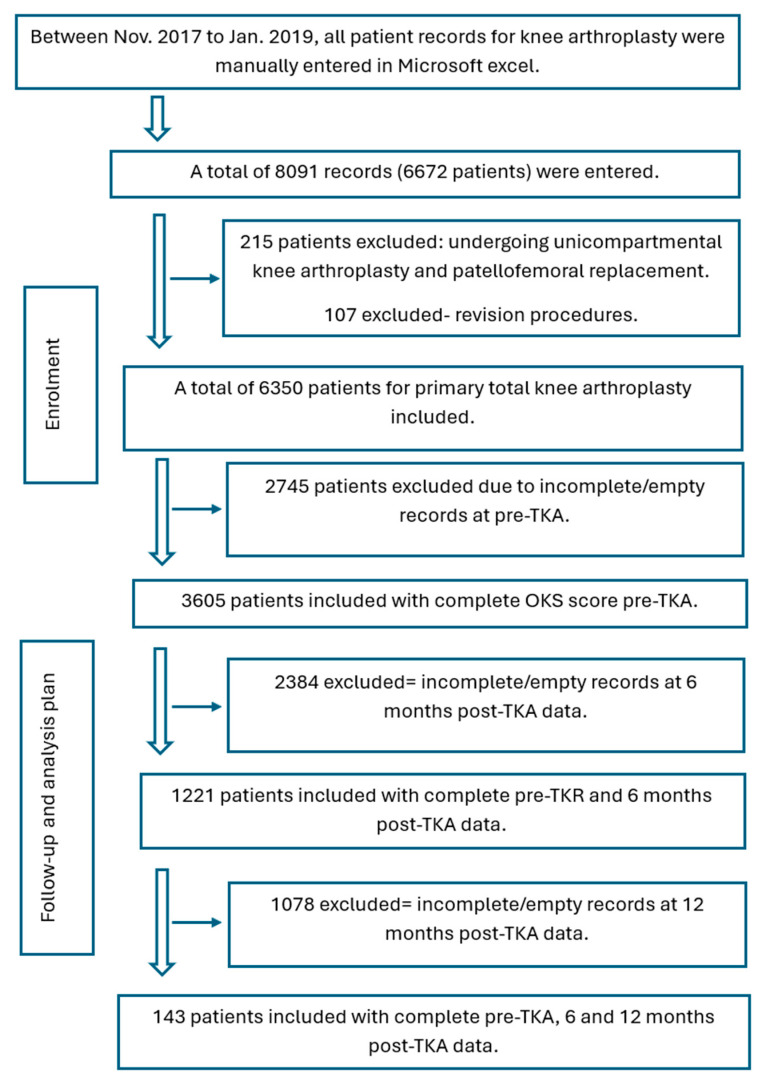
Participant flowchart in the UK.

After gaining ethical approval from the ethical committee in the NHS Health Research Authority (18/HRA/0168) and Salford University (HSR1617-137) a retrospective analysis was conducted for all patients undergoing primary TKA at Stepping Hill Hospital. The study was registered on ClinicalTrials.gov (NCT03132077). Participants were included in the analysis if they were scheduled for a primary unilateral total knee arthroplasty (TKA) due to end-stage knee osteoarthritis (Kellgren–Lawrence grade III and IV) and had a stable, well-managed medical condition. Eligibility was determined based on the availability of clinical records that met specific criteria: at least one preoperative assessment conducted within six months prior to surgery, available postoperative data at both six and twelve months after the procedure, and no more than two missing items in the questionnaire (Figure 1). When there were one or two missing scores, the missing values were filled by the mean value of all the remaining responses.

### 2.2. Study Design in KSA

**Figure 2:**
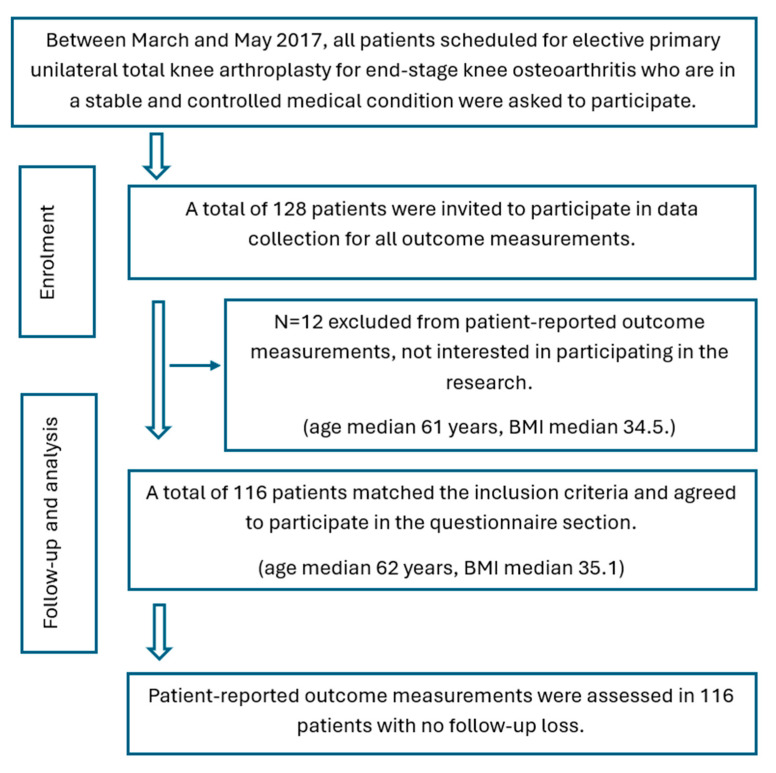
Participant flowchart in the KSA.

Due to the absence of available retrospective data, this study was conducted as a prospective cohort study to evaluate six- and twelve-month outcomes following primary total knee arthroplasty (TKA) in King Khalid University Hospital. Ethical approval was gained from the Ethical committee of Salford University (HSR1617-39) and the King Khalid University Hospital (E-17-2395). Additionally, the study was registered on ClinicalTrials.gov (NCT02998125). Patients with stable and well-controlled medical conditions who were scheduled for elective primary unilateral TKA due to advanced knee osteoarthritis (Kellgren–Lawrence grade III and IV) were asked to take part in the study (Figure 2).

Each eligible participant who provided informed consent was given an information sheet explaining the study objectives. Before enrolment, participants were fully informed of their rights. The OKS was collected from each participant before surgery, and six and twelve months post-TK.

### 2.3. Sample-Size Estimation

The required sample size was calculated using effect size and the standard deviation of difference (σ) from prior research [20,21]. This was performed with a statistical power of 0.95 (1 − β) and a significance level of α = 0.05. Based on these parameters, it was found that a sample size of 104 participants would be enough to calculate 4 point difference in OKS [20,21].

### 2.4. Surgical Management and Physical Rehabilitation

A midline incision with a medial parapatellar approach was performed on each patient. No intraoperative complications were reported. The post-surgical hospital stay lasted between five and six days. A standard posterior-stabilized total knee arthroplasty (TKA) design was used in the UK while high-flexion knee prostheses are utilized in the KSA. High-flexion knee designs facilitate greater flexion, allowing for activities such as kneeling, squatting, and crossed leg sitting—movements that are culturally and religiously significant. As a result, these prostheses are preferred over conventional posterior-stabilized TKA designs [22].

On the first postoperative day, the inpatient physical therapy program focused on promoting early mobilization, with patients engaging in weight-bearing or partial weight-bearing as tolerated. The rehabilitation protocol aimed to restore functional independence through a designed program comprising of bed exercises, range of motion (ROM) exercises, lower limb strengthening, gait training, and stair climbing practice.

The outpatient physiotherapy programs varied between the UK cohort and the KSA cohort. In the UK, rehabilitation typically involved three to six sessions, based on patient preference, with an emphasis on promoting self-confidence in performing knee ROM and lower limb strengthening exercises at home. In contrast, in Saudi Arabia, the outpatient physiotherapy protocol extended for at least four weeks, with patients attending three sessions per week. The rehabilitation regimen consists of progressive lower-limb ROM exercises, strengthening drills, and gait and balance training to ensure a comprehensive recovery.

### 2.5. Data Processing

The Oxford knee score (OKS) data was entered into the PSSS program in accordance with the 2015 OKS guidelines. Each item was rated on a scale from 0 to 4, where 0 indicated the worst outcome and 4 represented the best. The individual scores were added to generate a total score ranging from 0 (indicating the worst possible outcome) to 48 (indicating the best possible outcome) [9].

### 2.6. Statistical Analysis

The Mann–Whitney U test and chi-square analyses were used to compare demographic characteristics between patients from the UK cohort and the Saudi Arabia (KSA) cohort, as the data did not meet the normality assumption (*p* < 0.05). To assess differences in Oxford knee score (OKS) and mean scores across three postoperative time points, a two-way mixed ANOVA was conducted. This statistical test was selected to determine whether a significant difference existed between the groups over time. The analysis included a between-subjects group factor (UK, KSA), a within-subjects time factor (preoperative, 6 months, and 12 months post-TKA), and three covariates (age, gender, and body mass index (BMI)) to evaluate their potential influence on OKS outcomes [23].

## 3. Results

**Figure 3:**
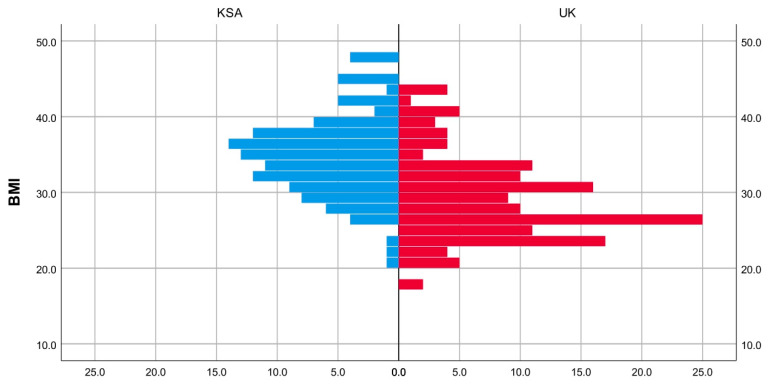
Population pyramid frequency: BMI by group.

**Figure 4:**
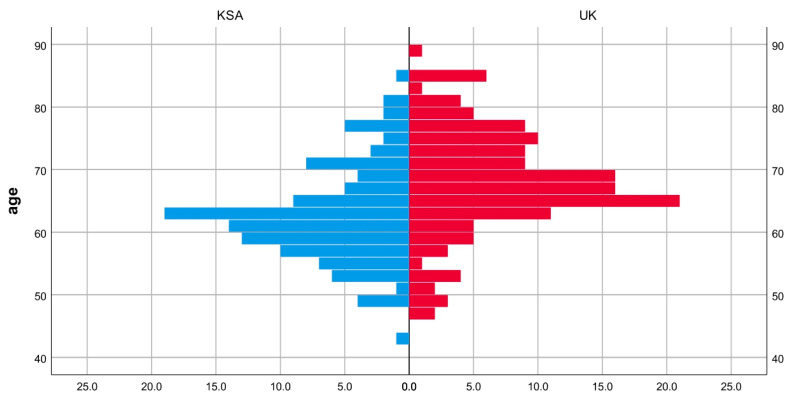
Population pyramid frequency: age by group.

The age distribution and BMI appeared to be almost similar between the group, as determined through visual inspection (Figure 3 and Figure 4). The medians for age and BMI were statistically significantly different; patient age was 5 years lower in the KSA cohort compared the UK cohort. Meanwhile, patient BMI was 7 points lower in the UK cohort compared to the KSA cohort (Table 1). A chi-square test revealed a significant difference in gender proportion between the two groups, *p* = 0.001. There was a higher proportion of males in the UK group than in the KSA group (37% vs. 17%) (Table 2).

The descriptive data for OKS score are summarized in Table 3 for each group at different time points. The assumption of sphericity was not meet for the two-way interaction, χ^2^ (2) = 97.2, *p* < 0.01. As a result, the ANOVA results were adjusted using the Greenhouse–Geisser correction. The time post-TKA and group (KSA vs. UK) significantly affected OKS scores (time: (1.33) = 12.23, *p* = 0.001; group: F (1.33) = 72.39, *p* = 0.001), while gender, age, and BMI showed no significant effect (*p* ≥ 0.05) at any time (Table 4).

**Figure 5:**
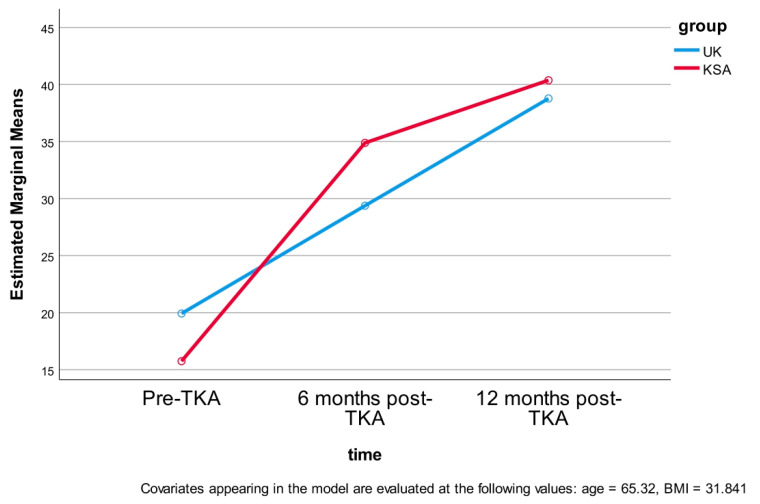
Estimated marginal means of OKS.

A multiple comparisons analysis was conducted to identify where significant differences occurred over time following TKA (pre, and six and twelve months post-TKA) and between groups (UK, KSA). At baseline, the UK cohort had a significantly higher OKS (19 ± 6) compared to the KSA cohort (15 ± 4), reflecting a 4-point difference. At six months post-TKA, the KSA cohort demonstrated a significantly higher OKS (34 ± 3) than the UK cohort (29 ± 5), with a 5-point difference. However, by twelve months postoperatively, the OKS between the UK (38 ± 3) and KSA (40 ± 1) groups revealed no statistically significant difference, with only a 1.8 difference (Table 5 and Figure 5).

## 4. Discussion

This current study aimed to compare the outcome of TKA between the KSA population and the UK population. While there were significant differences in patients’ demographic data and OKS at the baseline (before surgery) between both the UK cohort and the KSA cohort, by the end of twelve months after the surgery the scores were not significantly different.

In this current study, the median age of patients in the UK was older than the median age of patients in KSA, which also matched previous studies showing a broad range of patient ages in KSA [1,18,19]. The percentage of females was higher than males in the Saudi cohort, which is also consistent with previous studies [1,18,19].

The results revealed that Saudi Arabian patients had considerably lower OKS scores before the surgery, which may be explained by the significantly higher BMI compared to the UK group. This is consistent with previous research by Joshi and Pachore (2020) [24], which revealed substantial differences in OKS pre-TKA between different BMI groups. The extended waiting period for TKA surgery may also contribute to the significantly lower OKS at the baseline observed in KSA patients. The patients faced delays of up to 12 months for an initial orthopedic consultation, followed by another 12 to 24 months before undergoing surgery. In the United Kingdom, this waiting time is considerably shorter with an average of 6 months [25,26]. The age difference could be another factor which may affect the OKS at the baseline. Previous research has shown that the lower age group (<55 years old) shows significantly worse pain, function, and quality of life compared to older patients [27]. This may be related to higher activity levels and greater knee-related dysfunction among younger populations.

Several factors may explain the notable improvement in OKS scores for the Saudi group by the end of twelve months post-TKA in comparison to the scores pre-surgery. First, patients in Saudi Arabia were younger than the patients in the UK. However, the ANOVA analysis indicated that age did not statistically affect OKS outcomes over time. Previous studies have shown better long term improvement for young age groups after total knee replacement compared to older groups [27,28]. Another possible contributing factor could be the rehabilitation protocol itself. In KSA, patients received a minimum of twelve rehabilitation sessions, which may have encouraged greater commitment in exercise and supported improved recovery. Moreover, the use of high-flexion knee prosthesis design may have facilitated better performance in flexion-related activities, potentially improving responses to questions involving kneeling, as well as tasks such as climbing stairs, entering and exiting vehicles, or using public transportation. Interestingly, previous research has shown that higher flexion angle after surgery increases patient satisfaction, as reflected in the questionnaire [29].

This study has several limitations that should be recognized. First, the study design included a retrospective cohort from the UK and a prospective cohort from KSA. Both cohorts were engaged from single institutions, which may introduce institutional biases influencing demographic characteristics, rehabilitation protocols, surgical techniques, and functional outcomes. Moreover, this difference in study design may introduce potential biases linked to recall bias, data collection, and variability in documentation models. Additionally, although the sample size was sufficient for statistical analysis, a larger sample with more diversity may allow generalizability of the results with more certainty. Another limitation is the absence of detailed health risk and comorbidity data, such as diabetes, cardiovascular conditions, and musculoskeletal disorders, which may have affected recovery and therefore Oxford knee score (OKS) outcomes. Future studies should include more detailed patient health profiles to appropriately understand the impact of comorbidities on TKA recovery. Furthermore, the study did not account for ethnic and racial differences, which may also affect joint anatomy and surgical outcomes. Additionally, the difference in the study designs (prospective for the KSA cohort—retrospective for the UK cohort) may introduce inherent methodological biases. To lessen this effect, a two-way mixed ANOVA analysis was used adjusted for covariates (gender, age, BMI). However, residual confounding may persist, and direct comparison between cohorts with different designs should be interpreted carefully. Finally, cultural factors, such as differing expectations and perceptions of pain and functional recovery, were not extensively explored. Further qualitative and mixed-methods research design is required to evaluate how healthcare accessibility and cultural attitudes may influence patient experiences and long-term recovery after surgery.

## 5. Conclusions

This study highlights key differences in demographic characteristics and OKS between TKA patients in the UK and the KSA. While the Saudi cohort demonstrated lower baseline OKS scores, which is likely affected by several factors such as surgery waiting times, both groups demonstrated significant improvements postoperatively. By 12 months after the surgery, no significant difference remained in OKS, suggesting that any initial difference in recovery may diminish over the first year. These findings suggest that the healthcare system, and demographic and rehabilitation protocol differences between the cohorts may play a role in early TKA recovery. However, due to the observational design of this study, these relationships should be interpreted as indicative rather than causal, and further prospective research is needed to confirm these associations.

## Figures and Tables

**Table 1:** Demographic characteristics of patients from the UK cohort and the KSA cohort.

Variable	Cohort	Median	Interquartile Range	Maximum–Minimum	Mann–Whitney U Test	
					Standardized Test Statistic Z	*p*-Value
Age	UK Cohort	67	11	88–47	5.39	<0.01
	KSA Cohort	62	10	85–43		
Body mass index (BMI)	UK Cohort	28	7	44–18	7.56	<0.01
	KSA Cohort	35.1	6.6	48.5–20.6		

**Table 2:** Crosstabulation of gender distribution in the UK cohort and the KSA cohort.

Variable		Male	Female	Total	Chi-Square Test *p*-Value
UK Cohort	Count	53	90	143	<0.01
	Expected count	40.3	102.7	143	
	% within group	37.1%	62.9%	100%	
KSA Cohort	Count	20	96	116	
	Expected count	32.7	83.3	116	
	% within group	17.2%	82.8%	100%	
Total	Count	73	186	259	
	Expected count	73	186	259	
	% within group	28.2%	71.8%	100%	

**Table 3:** Oxford knee score (OKS) data for patients from the UK cohort and the KSA cohort following total knee arthroplasty.

Cohort	Time	Mean (SD)	Lower and Upper 95% Confidence Interval
			Lower Bound–Upper Bound
UK Cohort	Pre-surgery	19.4 (6.8)	18.16–20.65
	6 months post-surgery	28.9 (4.9)	28.03–29.92
	12 months post-surgery	38.4 (3.6)	37.68–39.07
KSA Cohort	Pre-surgery	15.2 (4.6)	14.33–16.03
	6 months post-surgery	34.7 (3.5)	33.9 2–35.21
	12 months post-surgery	40.2 (1.6)	39.94–40.52

**Table 4:** Two-way mixed ANOVA on Oxford knee score (OKS) changes post-TKA.

OKS Score Within Subjects’ Effect		Degree of Freedom	Mean Square	F	*p*-Value	Partial Eta Squared
Time	Sphericity assumed	2	159.98	12.23	<0.01	0.046
	Greenhouse–Geisser	1.29	246.34	12.23	<0.01	0.046
time * age	Sphericity assumed	2	8.68	0.66	0.52	0.003
	Greenhouse–Geisser	1.29	13.37	0.66	0.45	0.003
time * BMI	Sphericity assumed	2	21.85	1.67	0.19	0.007
	Greenhouse–Geisser	1.29	33.65	1.67	0.20	0.007
time * group	Sphericity assumed	2	947.03	72.40	<0.01	0.222
	Greenhouse–Geisser	1.29	1458.23	72.40	<0.01	0.222
time * Gender	Sphericity assumed	2	20.43	1.56	0.21	0.006
	Greenhouse–Geisser	1.29	31.45	1.56	0.22	0.006
time * group * Gender	Sphericity assumed	2	7.45	0.57	0.57	0.002
	Greenhouse–Geisser	1.29	11.48	0.57	0.49	0.002
Error (time)	Sphericity assumed	506	13.08			
	Greenhouse–Geisser	328.62	20.14			

*: Indicates an interaction term (e.g., time * age = interaction between time and age).

**Table 5:** Bonferroni multiple comparisons between the UK cohort and the KSA cohort at various time points.

Time Point	Mean Difference	Standard Error	*p*-Value	Lower and Upper 95% Confidence Interval
Pre-surgery	4.23	0.77	<0.01	2.70–5.76
6 months post-surgery	−5.60	0.57	<0.01	−6.73–−4.46
12 months post-surgery	−1.85	0.36	=0.063	−2.56–−1.15

## Data Availability

The raw data supporting the conclusions of this article will be made available by the authors on request.
